# Fibroin and fibroin blended three-dimensional scaffolds for rat chondrocyte culture

**DOI:** 10.1186/1475-925X-12-28

**Published:** 2013-04-08

**Authors:** Pratthana Chomchalao, Sutatip Pongcharoen, Manote Sutheerawattananonda, Waree Tiyaboonchai

**Affiliations:** 1 Faculty of Pharmaceutical Sciences, Naresuan University, Phitsanulok 65000, Thailand and the Center of Excellence for Innovation in Chemistry, Commission on Higher Education, Thailand; 2 Faculty of Medicine, Naresuan University, Phitsanulok 65000, Thailand; 3 Institute of Agricultural Technology, Suranaree University of Technology, Nakhon Ratchasima 30000, Thailand

**Keywords:** Fibroin, Collagen, Gelatin, Scaffolds, Chondrocytes

## Abstract

**Background:**

In our previous study, we successfully developed 3-D scaffolds prepared from silk fibroin (SF), silk fibroin/collagen (SF/C) and silk fibroin/gelatin (SF/G) using a freeze drying technique. The blended construct showed superior mechanical properties to silk fibroin construct. In addition, collagen and gelatin, contain RGD sequences that could facilitate cell attachment and proliferation. Therefore, in this study, the ability of silk fibroin and blended constructs to promote cell adhesion, proliferation and production of extracellular matrix (EMC) were compared.

**Methods:**

Articular chondrocytes were isolated from rat and cultured on the prepared constructs. Then, the cell viability in SF, SF/C and SF/G scaffolds was determined by MTT assay. Cell morphology and distribution were investigated by scanning electron microscopy (SEM) and histological analysis. Moreover, the secretion of extracellular matrix (ECM) by the chondrocytes in 3-D scaffolds was assessed by immunohistochemistry.

**Results:**

Results from MTT assay indicated that the blended SF/C and SF/G scaffolds provided a more favorable environment for chondrocytes attachment and proliferation than that of SF scaffold. In addition, scanning electron micrographs and histological images illustrated higher cell density and distribution in the SF/C and SF/G scaffolds than that in the SF scaffold. Importantly, immunohistochemistry strongly confirmed a greater production of type II collagen and aggrecan, important markers of chondrocytic phenotype, in SF blended scaffolds than that in the SF scaffold.

**Conclusion:**

Addition of collagen and gelatin to SF solution not only improved the mechanical properties of the scaffolds but also provided an effective biomaterial constructs for chondrocyte growth and chondrocytic phenotype maintenance. Therefore, SF/C and SF/G showed a great potential as a desirable biomaterial for cartilage tissue engineering.

## Background

Nowadays, millions of patients are suffering from cartilage defect caused by trauma, injury and age-related degeneration. Unfortunately, cartilage has a limited ability for self-repair due to its avascular, aneural and alymphatic characteristics. Moreover, current treatments for cartilage repair are unsatisfactory and rarely restore the structure of native cartilage [1,2]. A new approach as an alternative treatment for repairing, maintaining and improving tissue function is cartilage tissue engineering [3,4]. In this technique, biomimetic three dimensional (3-D) scaffolds are prepared as constructs for cartilage growth before implantation. To achieve this goal, biomimetic scaffolds with appropriate pore size, high mechanical properties, porosity and interconnecting pores are characteristics needed for polymeric scaffold design that provides temporary framework for supporting cell attachment, proliferation, differentiation and extracellular matrix formation [5-10]. Several techniques have been developed to fabricate 3-D scaffold using different synthetic and natural materials [11-15]. Among scaffold fabrication methods, freeze drying is widely utilized because of its simplicity and mild processing.

In recent years, SF, collagen and gelatin have been among the most extensively explored biomaterials for tissue engineering due to their biocompatibility, biodegradation and suitable mechanical properties. The biomaterial scaffolds made of these materials effectively provide temporary constructs for attachment and proliferation of fibroblasts, hepatocytes, chondrocytes, osteoblasts, and mesenchymal stem cells [16-23].

Silk fibroin (SF), a natural fibrous polymer produced by the silkworm, *Bombyx mori*, has been used as biomedical sutures for centuries. It is a protein mainly comprising of amino acids: glycine, alanine and serine that form a crystalline β-sheets in silk fibers, leading to its unique mechanical properties and hydrophobic domain structure. Additionally, silk fibroin has advantages of biodegradability, biocompatibility and low inflammatory response [24,25]. Therefore, it has a potential as natural biomaterial for biomedical applications. Collagen (C), one of the major components of the extracellular matrix, has been reported to inhibit the unwanted aggregation in fibroin scaffold during the preparative processes and produces scaffolds with high porosity [26,27]. It is a biodegradable natural protein with low antigenicity. Gelatin (G) is a partial hydrolysate of collagen. It has been widely used in surgery as a wound dressing and as biomaterial in the controlled drug delivery systems. In addition, both collagen and gelatin contain the amino acid sequence, arginine-glycine-aspartic acid (RGD), that stimulates cell attachment and protein expression in cells [28,29].

In our previous study, we successfully developed 3-D scaffolds prepared from SF, SF/C and SF/G using a freeze drying technique with methanol treatment [30]. These scaffolds exhibited sponge-like structure with homogeneous interconnecting pores and high water adsorption. We found that the mechanical properties of silk fibroin construct could be improved by blending with either collagen or gelatin. In addition, both of the blended constructs showed thicker pore wall and rougher surface than the SF scaffold suggesting that blended scaffolds should provide better environment for cell proliferation.

Apart from the superior physical properties of blended constructs to SF scaffolds, both additives, collagen and gelatin, contain RGD sequences that could facilitate cell attachment and proliferation. Thus, incorporating collagen and gelatin into SF scaffolds could improve chondrogenesis compared to pure SF scaffold. Therefore, in this study, rat chondrocytes were seeded on the three types of constructs; SF, SF/C and SF/G. Then, the characteristics of the constructs were compared based on their abilities to promote cell adhesion, cell proliferation and extracellular matrix (EMC) production.

## Materials and methods

### Materials

*Bombyx mori* raw silk yarns were purchased from Badint Thai-Silk Korat Co., LTD, Nakhonratchasima, Thailand. Bovine collagen was purchased from Fluka, USA. Type A Gelatin (~300 bloom) and Dulbecco’s Modified Eagle Medium were purchased from Sigma Chemical (St. Louis, MO, USA). Fetal bovine serum and penicillin/streptomycin solution were purchase from Gibco (California, USA). Thiazolyl blue tetrazolium bromide was purchase from Amresco® (Ohio, USA). Rabbit polyclonal antibody against aggrecan was purchased from Millipore Corporation (M2193, MA, USA). Mouse monoclonal antibody against type II collagen was purchased from Santa Cruze Biotechnology, Inc. (sc-52658, California, USA). Mouse monoclonal antibody against type I collagen was purchased from Abcam (ab6308, Cambridge, UK). Snakeskin pleated dialysis tube with MWCO at 10,000 Daltons was obtained from Thermo Scientific (Rockford, IL, USA). All other chemicals and solvents were of analytical grade.

### Preparation of 3-D silk fibroin based scaffolds

Three dimensional scaffolds of silk fibroin (SF), silk fibroin/collagen (SF/C), and silk fibroin/gelatin (SF/G) were prepared according to the procedure described in our previous study using a freeze-drying technique [30]. Briefly, SF solutions were prepared from 6% w/v silk fibroin aqueous solution. SF/C solution was prepared by mixing a 1% collagen solution with a 2% fibroin solution (25:75). The collagen solution was prepared by dissolving collagen in 5% v/v acetic acid [31] at 4°C and left overnight before use. To construct SF/G scaffolds, 4% gelatin aqueous solution was added to 6% fibroin solution (30:70). Then, the blending solutions were mixed with mild stirring for 20 min. Finally, the resulting solutions, SF, SF/C and SF/G, were transferred into polystyrene petri dishes and kept at −20°C overnight prior to lyophilization (PowerDry LL3000, Heto, USA). The dry porous sponges were removed from the dishes and treated with methanol for 30 min. Finally, methanol was evaporated at room temperature.

#### Physical characterization of the scaffolds

The morphology of porous 3-D scaffolds was investigated using a scanning electron microscopy (SEM, 1455VP, LEO Electron Microscopy Ltd., Cambridge, UK). The mean pore diameter of the scaffolds was determined by randomly measuring at least 30 pores from the SEM micrographs using an image analysis program called “ImageJ” (Java image processing program, downloaded from http://rsb.info.nih.gov/ij/index.html). The porosity of the prepared scaffolds was determined using liquid displacement method [32], employing hexane which easily penetrates the scaffolds without causing swelling or shrinkage. To determine the swelling property, the scaffolds were immersed in distilled water and the percentage water uptake was calculated from wet and dry weight of these scaffolds according the method from our previous study [30]. The mechanical property of each scaffold was measured at room temperature using an Instron-8872 (Instron Corporation, MA, USA.) equipped with a 0.25-kN load cell at a cross-head speed at 0.5 mm/min.

### Chondrocyte culture in 3-D scaffolds

Chondrocytes were isolated from articular cartilage of rats (male Sprague Dawley, 4–8 weeks) as approved by Naresuan University Animal Ethics Committee. The method for chondrocytes isolation was modified from Mohan et al. (2009). Briefly, cartilage specimens from the shoulder, hip and knee joints of rats were sliced and minced to small pieces and which were then washed three times in sterile 0.01 M phosphate buffered saline (PBS) pH 7.4. The cartilage matrix was sterile digested by 0.2% w/v collagenase II solution at 37°C, 5% CO_2_ for 1 hr. Finally, the cells were isolated by centrifugation at 1,500 rpm for 5 min and washed 3-times with serum-free Dulbecco’s Modified Eagle Medium (DMEM). The suspended chondrocytes were cultured in DMEM supplemented with 10% fetal bovine serum (FBS) and 1% of stock penicillin/streptomycin and maintained at 37°C, 5% CO_2_. Cell viability was determined by trypan blue dye exclusion assay. At confluency, articular chondrocytes from primary passage (P0) were further sub-cultured and expanded in tissue culture flasks.

Chondrocytes from a second passage (P2) were seeded on the SF, SF/C and SF/G scaffolds. Briefly, the sterilized scaffolds were shaped into a cylinder of diameter 10 mm and height 5 mm and washed twice with sterile PBS pH 7.4. Then, they were placed in 500 μl culture medium in a 24-well plate. After incubation overnight in the CO_2_ incubator, the medium was discarded and 50 μl of chondrocyte suspension containing ~10^6^ cells was seeded onto each construct. After 3 h, 500 μl of fresh culture medium was carefully added to each well and the cultures were maintained up to 28 days.

### Cell viability

The thiazolyl blue tetrazolium bromide (MTT) assay is widely used to assess cell viability, cell growth and toxicity based on changes in metabolic activity of cells [33]. Cell viability in the scaffolds was determined by MTT assay at specific time intervals of 0, 7, 14, 21 and 28 days. At the determined times, the samples were removed and transferred to new 24-well plates and 1 ml of serum-free medium containing 0.5 mg/ml MTT was added and cultured for a further 4 hours. The medium was then discarded and 2 ml of DMSO was added to dissolve the purple formazan crystals. The samples were shaken at 120 rpm for 30 min to ensure homogeneous dissolution of the formazan dye and then 200 μl of each sample was transferred to a 96-well plate. Optical density was measured at 595 nm using a microplate reader (Multimode detector DTX 880, Beckman Coulter Inc., Fullerton, USA.). SF, SF/C and SF/G scaffolds without chondrocyte seeding were used as control wells.

### Histological study

At the end of experiment, 28 days in culture, the constructs were collected and fixed in 10% neutral buffered formalin. Then, samples were embedded in paraffin, sectioned at 5 μm thickness, stained with hematoxylin-eosin (H&E) and examined under light microscope for revealing cell morphology and distribution.

### SEM examination of cell seeded scaffolds

After 28 days in culture, the scaffolds with attached cells were rinsed twice with 0.01 M PBS (pH 7.4), fixed with 3% glutaraldehyde in PBS for 3 hr and then rinsed twice with PBS for 10 min. The samples were dehydrated through graded ethanol solutions and air-dried for at least 10 hours. The scaffolds were then sectioned and mounted on aluminum stubs, vacuum sputter-coated with gold–palladium and examined under a scanning electron microscope.

### Immunohistochemical evaluation

The secretion of ECM by the chondrocytes in 3-D scaffolds was assessed by determination of chondrogenic makers, type II collagen and aggrecan, and compared with a fibroblastic maker, type I collagen. The presence of these markers in the 3-D scaffolds was tested with the relevant primary antibodies. Appropriate secondary antibodies using an immunoperoxidase tag Vectastain ABC kit (Vector Laboratories, Peterborough, UK) were employed according to the manufacturer’s instructions. Positive controls were performed using normal rat cartilage tissue for type II collagen and aggrecan and normal pig skin for type I collagen. Negative controls were run in parallel without the addition of primary antibodies.

### Statistical analysis

Statistical analyses of the data were carried out using a two-tailed unpaired Student’s *t*-test. Probability values (p) of less than 0.05 were considered statistically significant.

### Results

#### Physical properties of the 3-D silk fibroin based scaffolds

**Figure 1:**
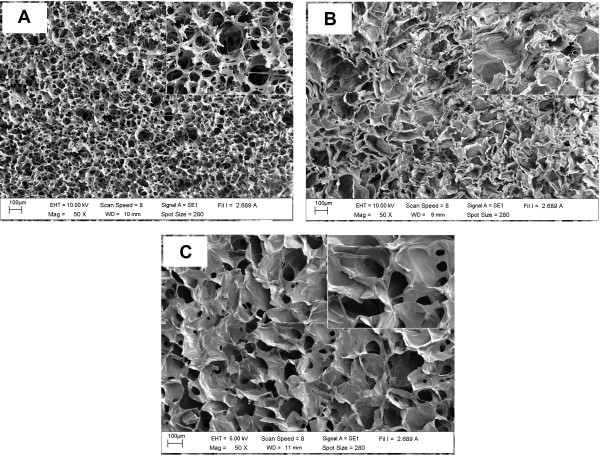
**SEM micrographs of prepared scaffolds after freeze-drying with methanol treatment.** (**A**) SF, (**B**) SF/C and (**C**) SF/G.

Three dimensional SF, SF/C and SF/G scaffolds were constructed using a freeze-drying technique with methanol treatment. A sponge-like structure of 3-D scaffold was obtained after lyophilization. SEM micrographs of all constructs illustrated a homogeneous porous structure with highly interconnecting pores, Figure 1. However, both types of blended scaffolds showed a thicker pore wall than SF scaffold. Interestingly, the rough surface suitable for cell attachment was observed in SF/C scaffold while SF/G scaffold exhibited a smooth surface similar to SF scaffold.

Mean pore size, porosity, water uptake ability and compressive property of different scaffolds were presented in Table 1. All constructs possessed high ability of water uptake, ~ 90%, with a suitable mean pore size for chondrocytes cultivation [34]. Nevertheless, blended scaffolds manifested a lesser porosity than SF scaffolds. As expected, SF/C and SF/G scaffolds showed a higher compressive property than SF scaffold. The effect of composition of scaffold on the physical properties of scaffold have been extensively discussed in previous study [30].

**Table 1:** Comparison of mean pore size, porosity, water uptake ability and mechanical property of different scaffolds

**Types of the scaffold**	**Mean pore size (μm) ± SD**	**% Porosity ± SD**	**% Water uptake ± SD**	**Compressive modulus (kPa) ± SD**
SF	65 ± 16	89 ± 0	92 ± 1	148 ± 12
SF/C	93 ± 21	61 ± 10	95 ± 1	1532 ± 697
SF/G	80 ± 28	61 ± 5	89 ± 3	364 ± 47

Values are average ± standard deviation (N = 3).

### In vitro cell viability and proliferation studies

**Figure 2:**
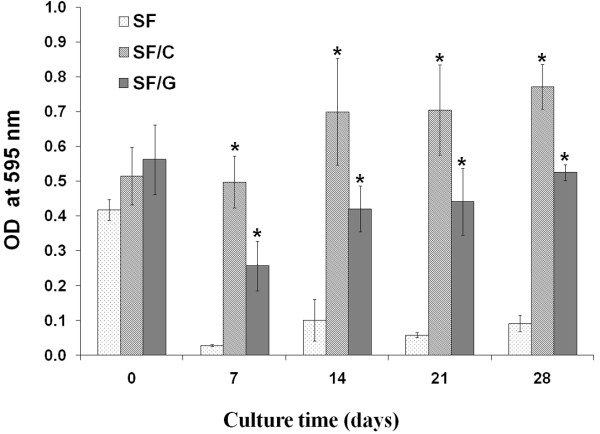
**MTT result after chondrocytes cultured in scaffolds for 0, 7, 14, 21, and 28 days. (****)** SF, **(****)** SF/C and **(****)** SF/G. *Significant differences are from SF scaffold at p < 0.05.

The MTT assay is a quantitative colorimetric assay used to access cell viability and proliferation. The purple formazan crystals created by metabolically active chondrocytes was detected by spectrophotometry at 595 nm. Thus, it is an indirect method for determining cell growth and proliferation. As shown in Figure 2, the OD values of blended scaffolds were significantly higher than those of SF scaffolds over cultivation time, at p < 0.05, indicating that cells were viable during the culture period. However, the OD value of SF scaffold was decreased after 7-day cultivation and remained stable over 28-day culture period suggesting that there might be cell death in the SF scaffold. It was worth noting that the OD values of SF/C and SF/G scaffolds were slightly decreased at the initial growth, during the first 7 days, and gradually increased afterward. This may be due to the rest or adaptation of the cells within a new environment required during the initial growth.

### Cell attachment and growth in 3-D scaffolds

**Figure 3:**
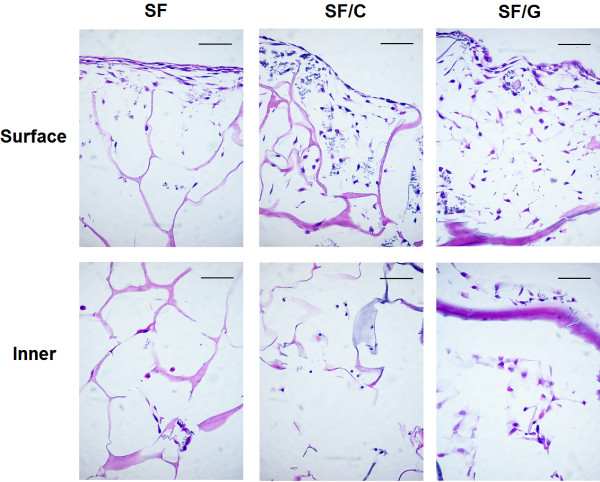
**Photomicrographs of chondrocytes growth on the surface and inner area of the scaffolds.** The chondrocytes were stained with hematoxylin and eosin. Original magnification x400, scale bar = 50 μm.

**Figure 4:**
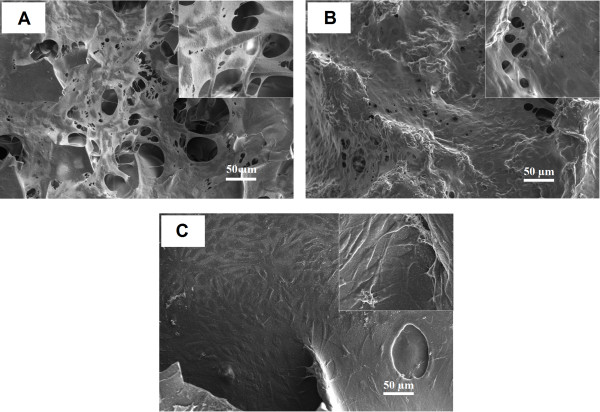
**SEM micrograph of chondrocytes growth on the surface area of the scaffolds.** (**A**) SF, (**B**) SF/C and (**C**) SF/G. Magnification x200.

**Figure 5:**
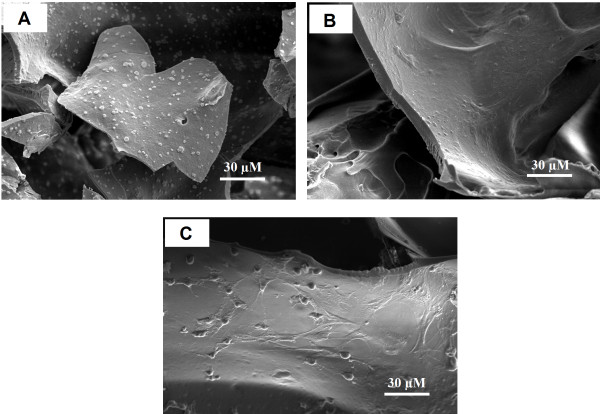
**SEM micrograph of chondrocytes growth in the inner area of the scaffolds.** (**A**) SF, (**B**) SF/C and (**C**) SF/G. Magnification x500.

The cell attachment and growth in 3 types of the constructs, SF, SF/C and SF/G scaffolds, were assessed by histological examination using hematoxylin-eosin staining. The morphology and distribution of chondrocytes on the surface and in the inner zone of the scaffolds after cultivation for 28 days were illustrated in Figure 3. Outer surface of all constructs were more highly populated with chondrocytes than the interior matrix. However, the number of cells that moved into the inner zone of SF/C and SF/G constructs appeared to be higher than those of SF scaffold. The cell morphology illustrated by H&E staining was consistent with SEM micrographs. As shown in Figure 4, the cell on the surface of constructs showed fibroblast-like morphology while inside constructs displayed a spherical shaped chondrocytes, Figure 5.

From SEM micrographs, Figure 4, all the cultured scaffolds look quite different compared to the raw native constructs, Figure 1, where the voids were unmistakable. For the SF scaffold, the SEM micrographs showed that large surface voids were still apparent (Figure 4A) but not so with the blended ones where the entire surface is covered with cells and the necessary ECM. The greater cell attachment and higher ECM secretion were observed on the SF/C and SF/G scaffolds than the SF scaffold. Interestingly, a high density of spherical shaped chondrocytes covered with ECM was displayed inside SF/C and SF/G scaffold, Figure 5.

### Immunohistochemistry of extracellular matrix

**Figure 6:**
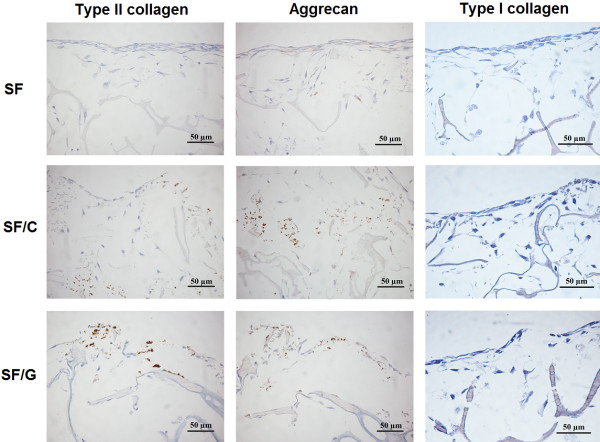
**Immunohistochemical staining of the sections for type II collagen, aggrecan and type I collagen.** Positive staining was indicated by light brown. Original magnification x400.

Type II collagen and aggrecan are differentiation markers of chondrocytes and considered as major components of cartilage tissue. The immunohistochemistry of 3-D constructs seeded with chondrocytes is shown in Figure 6. Scattered type II collagen staining was detected in both SF/C and SF/G scaffolds but was completely absent from SF scaffolds. Aggrecan staining was also detected in SF/C and SF/G constructs but also in the SF scaffold albeit at lower levels. In addition, the expression of type I collagen, fibroblast-specific collagen, was investigated to determine a sign of chondrocytes dedifferentiation. Immunohistochemical staining revealed a general absence of type I collagen staining in all types of constructs while pig skin stained heavily with the anti-type I collagen antibody (data not shown).

### Discussion

It is well known that a proper physical property of 3-D scaffolds is crucial for achievement in chondrocyte tissue engineering. Based on our previous study, three types of constructs prepared from SF, SF blended with collagen, and SF blended with gelatin were successfully developed. A sponge-like structure of 3-D scaffold was obtained with a homogeneous porous structure and highly interconnecting pores. All constructs possessed a high ability of water uptake with a suitable mean pore size for chondrocyte cultivation [34]. Nevertheless, the blended scaffold showed a superior physical property than SF scaffold. The thicker pore wall and higher compressive modulus observed with blended scaffolds offering greater compressive strengths required when implanted into joint capsules. In addition, the degradation rate of scaffold could be controlled by varying the type and amount of blended polymer [11,35]. Thus, this present study further investigated the superiority of SF/C and SF/G scaffolds on chondrocyte cultivation.

These results accord with those of Lv et al. [27] and Lu et al. [36] who demonstrated the potential of SF/C and SF/G for tissue engineering. Addition of collagen or gelatin to the SF scaffolds not only improved the mechanical properties of SF scaffold as previously reported but also greatly affected on the biological properties of chondrocyte cultivation. Both collagen and gelatin are biodegradable protein containing RGD sequence, a cell-recognition signal that promotes cell adhesion and proliferation. MTT assay confirmed that adding collagen or gelatin to the fibroin scaffold could promote chondrocyte survival and proliferation. This finding is in agreement for similar blends of SF/collagen [27] or of SF/gelatin [36] which could promote the proliferation of HepG2 and fibroblast cells, respectively. Although the SF scaffold offered the highest porosity and interconnecting pores which suitable for cell adhesion and migration, the cell death in the SF scaffolds was evident indicating unfavorable environment for cell survival compared to blended scaffolds.

Interestingly, as we found that SF scaffold is not suitable for cell survival. Wang et al. [37] reported that 3-D aqueous-derived silk fibroin provided a favorable environment for the proliferation of adult human chondrocytes (hCHs). One of the possible reasons was that in this present study, the cells were cultured in the DMEM medium without growth factors while Wang et al. reported culture cell in the medium with transforming growth factors β1 (TGF-β1) that is known to promote cell proliferation and adhesion. Another reason may be from the differences in microstructure of SF scaffold that provided less favorable condition for the growth of rat chondocytes. A smooth surface observed by SEM, hydrophobicity and the absence of the cell-recognition sequence of SF scaffold might be responsible factors. In addition, the greater hydrophilicity and faster degradation rate of blended SF scaffolds would be commensurate with chondrocyte migration and proliferation. Also noteworthy, cultivated cells prefer the rough surface of SF/C scaffold for better proliferation than the smooth surface of SF/G scaffold [27].

In general, the mature chondrocytes are characterized by a rounded morphology and the production of cartilage extracellular matrix such as type II collagen and aggrecan. However, articular chondrocytes isolated from rat were limited in number. Therefore, chondrocyte expansion in monolayer condition was necessary before cultivation on the tested scaffolds. Unfortunately, the monolayer expansion causes dedifferentiation of chondrocytes as shown by a fibroblastic morphology accompanied by loss of type II collagen expression [38-41]. Fibroblasts are characterized by flattened shapes and the expression of type I collagen. Thus, re-differentiation and restoration of the chondrocytic phenotype during the cultivation period is crucial.

After cultivation on different types of constructs for 28 days, chondrocyte morphology and secretion of ECM were determined. Regardless of the types of materials used, chondrocytes found on the surface of constructs exhibited fibroblast-like cells with relatively homogeneous ECM accumulation. Nevertheless, immunohistochemical studies confirmed re-differentiation and maintenance of the chondrocytic phenotype during cultivation in all types of constructs [42-44]. This was evident from an expression of type II collagen and aggrecan which are chondrocyte specific proteins, as well as a failure to detect type I collagen expression that is fibroblast specific protein. The change into fibroblast-like cell is commonly found when culturing in 2-D environment. This might be due to the fact that the surface of the construct is not 3-D, but is a 2-D-like environment as in the culture dish. A recent study has suggested the use of heat inactivated allogeneic serum may be useful in protecting against dedifferentiation of the chondrocytes [45].

Clearly, the results confirmed the superiority of collagen or gelatin blends on chondrocyte cultivation in 3-D construct over SF alone. Both of the blended constructs demonstrated ECM enveloping the surface more than SF scaffold. In addition, chondrocytes cultured in SF/C and SF/G scaffolds were effectively moved into the inner zone and most of the cells inside these areas displayed spherical shape with ECM secretion. In contrast, chondrocytes rarely found in the inner zones of SF constructs corresponding to the MTT assay results. Among 3 types of scaffolds tested, SF scaffold manifested the most hydrophobicity and the lowest degradation rate that could limit cell-cell interaction and migration resulting in cell death, while the blended scaffolds possessed hydrophilicity and faster degradation rate that could help in promoting cell-cell interaction and ECM secretion.

### Conclusions

While varieties of biomaterial scaffolds have been fabricated and proposed for use in cartilage tissue engineering, few studies have compared the biological response of chondrocytes in different scaffolds. This study prepared three types of silk fibroin-based scaffolds by freeze drying method and found the significant difference in cell proliferation and extracellular matrix formation among SF based constructs tested. In conclusion, based on the findings in this study, SF-based collagen and gelatin hybrid scaffolds can be served as another possible biomaterial to create a 3-D scaffold with an adequate strength to support the growth of chondrocytes. The RGD signal and hydrophilicity play a key role in cell attachment, proliferation and production of ECM. Therefore, SF/C and SF/G scaffolds have great potential for cartilage tissue engineering application. Nevertheless, *in vivo* study in animal model remains to be further investigated.

## Abbreviations

3-D: Three dimensional; SF: Silk fibroin; SF/C: Silk fibroin/collagen; SF/G: Silk fibroin/gelatin; RGD: Arginine-glycine-aspartic acid; ECM: Extracellular matrix.

## Competing interests

The authors declare that they have no competing interests.

## Authors’ contributions

WT participated in research design, discussion of result and final revision. PC contributed in research design, performed the experiments, analysis and discussion of result, and manuscript preparation. SP and MS aided in the study design, discussion and manuscript drafting. All authors read and approved the final manuscript.
